# Boron and Gadolinium Loaded Fe_3_O_4_ Nanocarriers for Potential Application in Neutron Capture Therapy

**DOI:** 10.3390/ijms22168687

**Published:** 2021-08-13

**Authors:** Ilya V. Korolkov, Alexandr V. Zibert, Lana I. Lissovskaya, K. Ludzik, M. Anisovich, Artem L. Kozlovskiy, A. E. Shumskaya, M. Vasilyeva, Dmitriy I. Shlimas, Monika Jażdżewska, Beata Marciniak, Renata Kontek, Dorota Chudoba, Maxim V. Zdorovets

**Affiliations:** 1 The Institute of Nuclear Physics, Ibragimov Str. 1, Almaty 050032, Kazakhstan; alexandr.zibert@bk.ru (A.V.Z.); ms.defrance@mail.ru (L.I.L.); kozlovskiy.a@inp.kz (A.L.K.); Shlimas@mail.ru (D.I.S.); mzdorovets@inp.kz (M.V.Z.); 2 Engineering Profile Laboratory, L.N. Gumilyov Eurasian National University, Satpaev Str. 5, Nur-Sultan 010008, Kazakhstan; 3 Department of Physical Chemistry, University of Lodz, 90-236 Lodz, Poland; 4 Frank Laboratory of Neutron Physics, Joint Institute for Nuclear Research, Dubna 141980, Russia; mojaz@amu.edu.pl (M.J.); scientific_secretary@nf.jinr.ru (D.C.); 5 Republican Unitary Enterprise, Scientific-Practical Centre of Hygiene, 220012 Minsk, Belarus; m_anisovich@mail.ru (M.A.); m_anisovich@gmail.ru (M.V.); 6 The Institute of Chemistry of New Materials, National Academy of Sciences of Belarus, 220072 Minsk, Belarus; lunka7@mail.ru; 7 Faculty of Physics, Adam Mickiewicz University, 61-614 Poznan, Poland; 8 Laboratory of Cytogenetics, Faculty of Biology and Enviromental Protection, University of Lodz, 90-231 Lodz, Poland; beata.marciniak@biol.uni.lodz.pl (B.M.); renata.kontek@biol.uni.lodz.pl (R.K.); 9 Department of Intelligent Information Technologies, Ural Federal University, Mira Str. 19, Ekaterinburg 620002, Russia

**Keywords:** superparamagnetic Fe_3_O_4_ nanoparticles, carborane, gadolinium, targeted drug delivery, NCT, cytotoxicity

## Abstract

In this article, a novel method of simultaneous carborane- and gadolinium-containing compounds as efficient agents for neutron capture therapy (NCT) delivery via magnetic nanocarriers is presented. The presence of both Gd and B increases the efficiency of NCT and using nanocarriers enhances selectivity. These factors make NCT not only efficient, but also safe. Superparamagnetic Fe_3_O_4_ nanoparticles were treated with silane and then the polyelectrolytic layer was formed for further immobilization of NCT agents. Fourier-transform infrared spectroscopy (FTIR), X-ray diffraction (XRD), energy dispersive X-ray (EDX), ultraviolet–visible (UV-Vis) and Mössbauer spectroscopies, dynamic light scattering (DLS), scanning electron microscopy (SEM), vibrating-sample magnetometry (VSM) were applied for the characterization of the chemical and element composition, structure, morphology and magnetic properties of nanocarriers. The cytotoxicity effect was evaluated on different cell lines: BxPC-3, PC-3 MCF-7, HepG2 and L929, human skin fibroblasts as normal cells. average size of nanoparticles is 110 nm; magnetization at 1T and coercivity is 43.1 emu/g and 8.1, respectively; the amount of B is 0.077 mg/g and the amount of Gd is 0.632 mg/g. Successful immobilization of NCT agents, their low cytotoxicity against normal cells and selective cytotoxicity against cancer cells as well as the superparamagnetic properties of nanocarriers were confirmed by analyses above.

## 1. Introduction

Cancer disease remains the second leading cause of death worldwide due to it aggressiveness and treatment difficulty. In America, the number of estimated cases in 2019 is 1,762,450. One of the traditional methods to treat cancer is chemotherapy, but because of its damage to normal cells surrounding the tumor it can lead to consequences, such as cardio toxicity, neurotoxicity, infertility, nephropathy, and chronic liver damage [1,2,3]. Over the last two decades, the attention paid to cancer disease has increased, and plenty of theoretical and engineering research has been conducted. As a result, new, less damaging methods, such as radiation therapy, immunotherapy, cancer hyperthermia, microbial-based cancer treating systems, have appeared [4,5,6,7,8]. Even though radiotherapy has shown its effectiveness, kinds of tumor such as adenocarcinomas, sarcomas, carcinomas, and gliomas are considered to be radioresistant and demand special approaches [9]. For example, neutron capture therapy (NCT) has shown promising results in treating glioblastoma and some other radioresistant tumors. This method can be characterized by its high selectiveness and the effectiveness of treatment [10].

NCT is founded on the delivery of drugs labeled by specific radioisotopes, for instance, ^10^B and ^157^Gd, directly to the tumor and irradiating it by thermal neutron flux. As the result of the neutron-capture reaction, α-particles and Auger–Coster–Kronig electrons have a short pathlength in tissues (less than 10 μm for α-particles) and high linear energy transfer (LET). These particles damaging DNA, so the apoptosis process in the cancer cells begins. If NCT agents are selectively delivered to cancer cells, because of the short pathlength of particles, damage to normal tissues is minimized. Due to the high LET, NCT provides increased relative biological effectiveness and a lower oxygen enhancement ratio [11,12]. Clinical trials were provided using the boron-labeled radioisotopes, such as 4-borylphenylalanine (BPA) and sodium mercaptoundecahydro-closo-dodecaborate (BSH). These trials have shown the necessity to develop more selective delivery methods [13,14]. Later delivery methods use active and passive targeting. Passive targeting is based on the enhanced permeability and retention (EPR) effect (ability of nanoparticles to penetrate through vascular architecture). Active targeting is based on the attaching to the molecule or nanocarrier special antibody or ligand in order to point them to tumor cells [15]. Nanostructures used for the delivery of NCT agent include boron nitride nanotubes, liposomes, dendrimers, polymeric nanoparticles, and others [16,17,18,19].

In addition, the delivery of agents can be provided by immobilizing to the magnetic nanoparticles and manipulating the external magnetic field [20,21,22,23,24]. Iron oxide nanoparticles, spinel ferrites nanoparticles and core–shell composite nanoparticles might be used for these purposes due to their magnetic properties [25,26,27].

Zhu et al. declare the concentration of delivered boron through magnetic nanoparticles to be 51.4 μg/g [28]. A higher concentration of NCT agents can be achieved by covering the magnetic nanoparticle with polymer to make the surface’s structure branched [29]. Gd ions can be immobilized via chelate complexes, such as Gd-pentaacetate (Gd-DTPA) [30]. Authors [11,31,32] have shown benefit in using both of boron and gadolinium compounds due to the added diagnostic properties and synergetic effect in the neutron capture reaction. For example, it is declared that adding Gd-DTPA has decreased the survival of cancer cells by 80–90%. Nevertheless, the methods of immobilization of these two types of NCT agents to nanoparticles have not yet been described.

In this paper, we represent the novel result of the simultaneous immobilization of gadolinium and boron-based NCT agents onto Fe_3_O_4_ nanoparticles via moderate and biocompatible polyelectrolyte poly(acrylic acid) (PAA)/poly(allylamine) (PALAm) formation.

## 2. Results and Discussion

**Figure 1:**
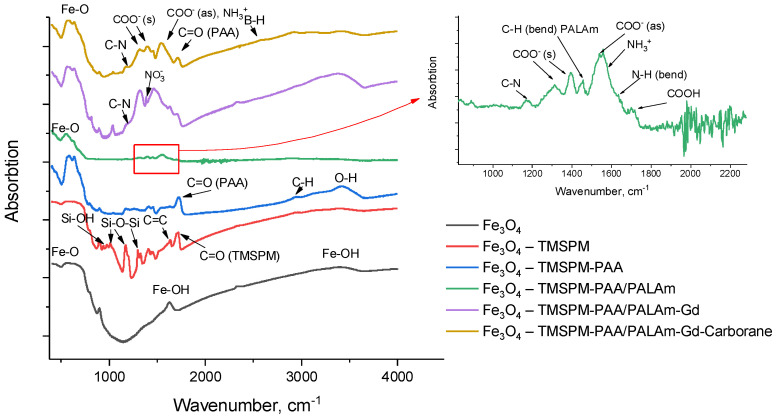
FTIR spectra of Fe_3_O_4_, Fe_3_O_4_-TMSPM, Fe_3_O_4_-TMSPM-PAA, Fe_3_O_4_-TMSPM-PAA/PALAm, Fe_3_O_4_-TMSPM-PAA/PALAm-Gd, Fe_3_O_4_-TMSPM-PAA/PALAm-Gd-Carborane.

Fe_3_O_4_ were obtained by the chemical coprecipitation with ammonia hydroxide, and their average size was 27 nm according to SEM. Characteristic peaks are observed for FTIR spectra at 575 cm^−1^ for Fe-O-Fe bonds and 1630 cm^−1^, 3375 cm^−1^ of which are related to Fe-OH (Figure 1) [33].

**Figure 2:**
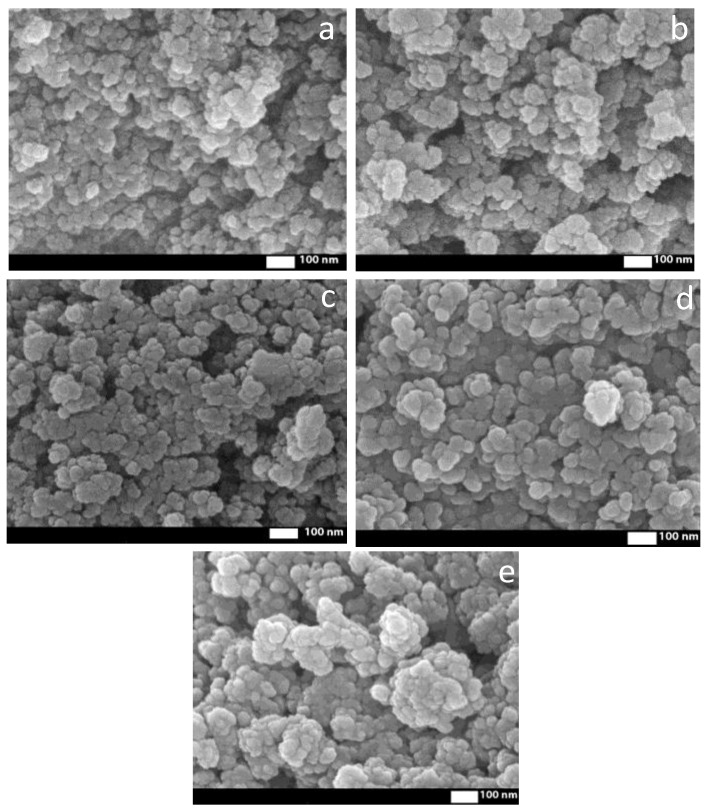
SEM images of Fe_3_O_4_ (**a**), Fe_3_O_4_-TMSPM (**b**), Fe_3_O_4_-TMSPM-PAA (**c**), Fe_3_O_4_-TMSPM-PAA/PALAm (**d**), Fe_3_O_4_-TMSPM-PAA/PALAm-Gd (**e**).

**Scheme 1:**
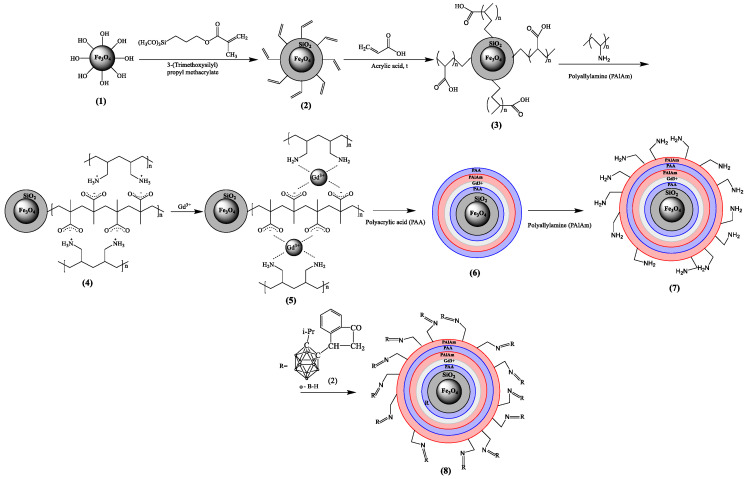
Scheme of Fe_3_O_4_ modification and carborane and gadolinium ion immobilization.

Then, the surface was functionalized according to Scheme 1 with C=C double bonds via the polycondensation reaction with 3-(trimethoxysilyl)propyl methacrylate (TMSPM). It is based on the removal of CH_3_O- from alkoxysilane and attaching it to the OH group of Fe_3_O_4_ nanoparticles. Si-O-Si and C=C bonds are observed at 1170, 1012 cm^−1^ and 1635 cm^−1^, respectively; peak at 1296 cm^−1^ can be also attributed to the stretching vibrations of Si-O-Si bonds [27]; the small peak at 937 cm^−1^ can be related to Si-OH, indicating the incompleteness of the reaction [34,35]. The peak at 1723 cm^−1^ is related to C=O bonds. An average size increasing up to 28 nm has been observed at SEM (Figure 2). An EDX analysis has shown the presence of Si in elemental composition.

The next stage of modification is the thermoinitiated graft polymerization of acrylic acid (AA). N=N bonds in the initiator, 2,2′-Azobis(2-methylpropionamidine) dihydrochloride (ABAP), are being opened with the creation of two radicals. The reaction of radicals with the C=C groups assists the graft polymerization of AA to the surface of functionalized nanoparticles. The time of the reaction, temperature, concentration of initiator and monomer, and chosen solvent can determine the amount of grafted polymer. The optimal conditions of this reaction were found by changing these parameters. The described nanoparticles can be well dispersed in ethanol, but ABAP is poorly soluble in pure ethanol; that is why 50% ethanol aqueous solution have been chosen. The control of the reaction was provided by FTIR spectroscopy; PAA can be characterized by C-H stretching bond at 2950 cm^−1^, carboxyl groups peak (1725 cm^−1^) [36]. Intensity ratio of carboxyl groups peak to Fe-O-Fe peak (575 cm^−1^) can be an indication of the amount of grafted PAA. The results are presented in Table 1. It is observed that amount of grafted PAA increases with time and decreases with the higher concentrations of PAA. This relates to the phenomena of homopolymerization. Though a C=O double bonds peak was also observed at the previous step, this one is related precisely to PAA, otherwise it would have reduced in the same way as the Si-O-Si (1170 cm^−1^) peak. Another argument for the presence of PAA can be made because of the increased O-H peak (3319–3573 cm^−1^).

SEM confirms the appearance of polymeric layer due to size increasing to 29 nm (Figure 2c). The concentration of grafted COOH groups was examined by colorimetric assay using toluidine blue: 174.61 μM/g. This additionally confirms the grafting of PAA to the surface of nanoparticles.

After, the polyelectrolyte layer was formed by the precipitation of positively charged PALAm chains to the negatively charged PAA chains. The deposition of the layer can be confirmed by the characteristic peaks at 1178 cm^−1^, 1452 cm^−1^, 1635 cm^−1^ and 3340 cm^−1^ for C-N stretching, C-H and N-H bending vibration, O-H/N-H stretching vibration, respectively [34]. Additionally, the broad peak at 1500–1600 cm^−1^ can be attributed to ν_as_ COO- bonds and NH_3_^+^, and bands attributed to the vibrations of ν_s_ COO^−^ (1390 cm^−1^) and ν_s_(CO) + δ(OCO) (1310 cm^−1^) [25], confirming the formation of polyelectrolyte. The average size of particle with polyelectrolyte layers is 35 nm (Figure 2d) according to SEM. This polyelectrolyte, due to its carboxyl and amino groups, is able to form chelates with gadolinium ions [37,38]. The concentration of amino groups was measured by colorimetric assay using acidic orange and equal to 5.34 μM/g. After immobilization of PALAm, EDX analysis has detected nitrogen and chlorine (Table S1).

The next step of modification is the immobilization of Gd ions. FTIR spectrum has shown peaks that related to NO_3_^−^ at 1386 cm^−1^ [39], and a shift of COO- groups was observed at 1319 and 1399 cm^−1^. The appearance of a nitrate ions probably indicates that first a complex of gadolinium with a polymer containing nitrate ions is formed, but then, after the formation of the second polyelectrolyte layer, nitrate ions were not detected. Thus, a more stable gadolinium complex with PAA and PALAm is formed, and the nitrate ions were washed out. The average size of Fe_3_O_4_-TMSPM-PAA/PALAm-Gd nanoparticles was 33 nm (Figure 2d). Additionally, EDX analysis confirmed the immobilization of Gd by 2.9% (Table S1).

After that, a second layer of polyelectrolyte was immobilized for attaching the 3-(isopropyl-o-carboranyl) hydrindone. EDX shows the grown percentage of carbon and nitrogen up to 43.6% and 12%. Then, 3-(isopropyl-o-carboranyl)hydrindone was attached to modified Fe_3_O_4_ nanocarriers. FTIR spectroscopy detected a new peak at 2600 cm^−1^ related to B-H stretching modes [40]. Additionally, boron was detected in the EDX analysis and its content was 10.4 at.%. It should be noted that the determination of concentration for light elements such as boron can be overestimated by EDX analysis, since oxygen and carbon peaks can overlap with the boron line. The amount of boron and gadolinium in the final sample was also estimated by laser-induced breakdown spectroscopy (LIBS); the amount of B is 0.077 mg/g (7 µM/g) and the amount of Gd is 0.632 mg/g (4 µM/g). It was previously found that the concentration of amino groups before the immobilization of gadolinium ions is 5.34 μM/g, which is comparable to the content of gadolinium in the final sample.

**Figure 3:**
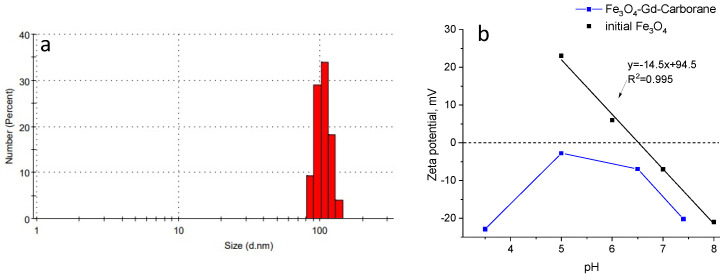
Size distribution of Fe3O4-TMSPM-PAA/PALAm-Gd-Carborane (**a**) and zeta-potential as function of pH (**b**).

The size was examined by DLS analyses (Figure 3). By DLS analyses, the average hydrodynamical size is equal to 110 nm that differs from SEM results. However, it should be noted that size distribution obtained from SEM shows dry state of the sample, whereas DLS allows the sample to be observed in the solvated state where solvent molecules associate with particle [41]. Moreover, the system can exhibit the tendency to aggregation due to the attractive particle–particle interactions. Figure 3b shows a graph of the dependence of pH on zeta potential. The charge of initial Fe_3_O_4_ reverses from positive to negative at pH around 6.3, which can be considered an isoelectric point. The surface of Fe_3_O_4_ nanoparticles can form positive (Fe–OH_2_^+^) and negative (Fe–O^−^) surface charges. Fe_3_O_4_-TMSPM-PAA/PALAm-Gd-Carborane are negatively charged at pH values from 3.5 to 7.5. This can indicate that negatively charged carboxylic groups of grafted PAA are in excess.

**Figure 4:**
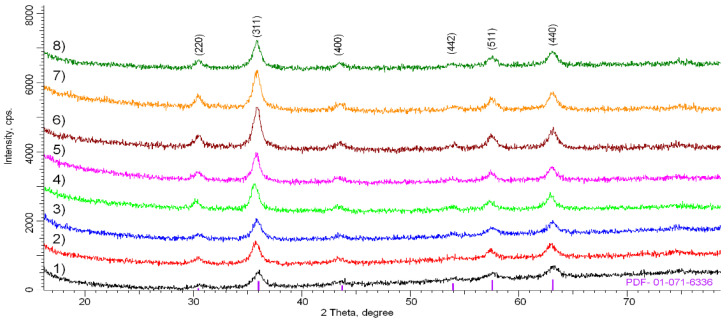
X-ray diffraction of Fe_3_O_4_ at different stages of modification: Fe_3_O_4_ (1), Fe_3_O_4_-TMSPM (2), Fe_3_O_4_-TMSPM-PAA (3), Fe_3_O_4_-TMSPM-PAA/PALAm (4), Fe_3_O_4_-TMSPM-PAA/PALAm-Gd (5), Fe_3_O_4_-TMSPM-PAA/PALAm-Gd-PAA (6), Fe_3_O_4_-TMSPM-PAA/PALAm-Gd-PAA/PALAm (7), Fe_3_O_4_-TMSPM-PAA/PALAm-Gd-PAA/PALAm-Carborane (8).

Figure 4 shows the X-ray diffraction patterns of the samples, depending on the stage of modification. The general view of the diffraction patterns is typical for polycrystalline samples with an average degree of crystallinity due to the nanoscale particle size, as well as the presence of disordered regions characteristic of oxide nanoparticles obtained using the wet chemical method. Using a full-profile analysis, it was determined that the position and intensities of the peaks are characteristic of the cubic phase of magnetite Fe_3_O_4_ with broadened reflections and altered positions of the interplanar distances, which indicates the disordering of the structure. In this case, depending on the stage of modification, partial ordering and an increase in the intensity of diffraction reflections are observed, which can be caused by both a change in stoichiometry and a change in the vacancy and defect structures. Intensity fluctuations in the 2θ = 15–28° region can be associated with amorphous-like reflections characteristic of polymer compounds and silanes used for modification.

These peaks are related to the Fe_3_O_4_ phase and characterized by Miller indexes (220) and (442). As the literature shows, nanostructural Fe_3_O_4_ is distinguished by its high content of disordered regions due to the big concentration of vacancy defects in structure [42,43]. In addition, the characterization of these structures by methods of roentgen diffraction was obstructed due to the hashing of peaks characteristic for Fe_3_O_4_ and FeO·Fe_2_O_3_ and some nonstoichiometric phases that can appear while the synthesis process. In some works [29,44], for a proper characterization and estimation of the phase content of Fe_3_O_4_ nanoparticles synthesized using the wet chemical method, a combined roentgen diffraction and Mössbauer spectroscopy method was used. This method is more sensitive to the division of different phases contribution because values characteristic for hyperfine fields are significantly different.

Table 2 shows the results of changes in structural parameters calculated based on the analysis of X-ray diffraction patterns. Stoichiometry was calculated according to [45,46]. Partial oxidation of iron (II) during modification can occur by oxygen dissolved in solvents at each stage, also oxidation is possible during drying and storage. As a result, non-stoichiometric Fe_3-δ_O_4_ can be formed where δ can range from zero (stoichiometric magnetite) to 1/3 (completely oxidized). As can be seen from XRD analysis, stoichiometry ranges from Fe_2.88_O_4_ for initial nanoparticles to Fe_2.71_O_4_ for final nanoparticles with payloads.

Figure S1 shows the Mössbauer spectra of Fe_3_O_4_ at different stages of modification. The general view of the obtained spectra indicates that the use of various types of modifications has a significant effect on the magnetic properties of nanoparticles, as well as the degree of their magnetic ordering.

The studied spectra of nanoparticles were interpreted using a model that includes two sextets characteristic of the structure of magnetite Fe_3_O_4_ of the AB_2_O_4_ type, in which iron ions are located in the A-sublattice in the tetrahedral, and in the B-sublattice—in the octahedral environment of oxygen atoms, as well as one quadrupole doublet characteristic of impurity inclusions or disordering regions. In this case, a strong broadening of the lines of partial spectra indicates the presence of disordering regions in the structure and nonstoichiometry of nanoparticles, which, in the case of ideal magnetite, should correspond to the ratio of the spectral intensities in the B and A sublattices as 2:1. Any deviations from this ratio mean the presence of additional vacancies in the structure, excess or lack of oxygen. Data extracted from the Mössbauer spectra are presented in Table 3. 

As can be seen from the presented data, the values of the hyperfine magnetic fields correspond to the disordered structure of magnetite Fe_3_O_4_ of the AB_2_O_4_ type, and the ratio of the intensities of the partial spectra I_B-sublattice_/I_A-sublattice_ indicates a strong deviation from stoichiometry. This deviation from stoichiometry can be explained by the presence in the structure of cation vacancies associated with an excess of oxygen in the structure, which occupies octohedral positions; as a result, the intensity of these spectral lines is higher.

The same changes were observed in Ref. [47], where the authors use the microemulsion-methods-obtained magnetic nanoparticles with different polymeric additions, which, according to the Mössbauer spectroscopy data, led to the deviation from stoichiometry, changes in the corrosion and oxidation mechanisms and concentration of defects. In this case, an asymmetric spectra shape is associated with stoichiometry changes and A- and B- iron ions concentration in sublattice. Additionally, according to the received data, hyperfine field magnitude values for A- and B- sublattices are well correlated with data from the literature and characteristic for the magnetite phase of Fe_3_O_4_ [48,49]. At the same time, in the case of modification by silanes and polymers, vacancy positions can also be occupied by silicon, carbon, or nitrogen ions, which leads to an additional broadening of the lines of the partial spectra, as well as an increase in the contribution characteristic of the quadrupole doublet.

The magnetic properties of magnetic nanoparticles with different functional surfaces are diverse (Figure S2). Physical quantities, such as magnetization at 1T, coercivity (He), remanent magnetization (Mr), quadrant of hysteresis loop (K) which are calculated from hysteretic loops, are shown in Table S2. The magnetic parameters of initial samples have values specific for ferromagnetic magnetite nanoparticles with low coercivity, which are distinctive for nanosized samples [50]; this was also confirmed with SEM. The magnetization of magnetite nanoparticles is being reduced alongside the functionalization process due to the addition of a nonmagnetic phase to the composite content. This corresponds with the elemental analysis shown in Table S1.

The reduction in remanent magnetization for Fe_3_O_4_-TMSPM, Fe_3_O_4_-TMSPM-PAA, and Fe_3_O_4_-TMSPM-PAA/PALAm samples indicates the magnetite nanoparticles’ surface magnetic-shell-altering processes occur as a result of the formation of layers and also the reduction in particle dipole–dipole interaction due to the increasing size. The samples’ coercivity reduction might be explained by the partial magnetic core destruction. Doping functionalized nanoparticles with gadolinium ions leads to almost twice the growth of magnetic parameters due to the increase in the degree of magnetic core crystallinity and crystallite size (Table 2) [51].

Thus, the determined magnetic parameters indirectly indicate successful magnetite nanoparticles functionalization with polymeric complex and the formation of the “core-shell” structure. While attaching new functionality, doped gadolinium ions lead to the altering of the crystalline structure of magnetite; as a result, the magnetic properties change.

Magnetic nanoparticles with different structures, sizes and stoichiometries are being reviewed for biomedical applications, such as MRI, drug delivery, magnetic hyperthermia and theranostics in the literature. The magnetic parameters (coercivity, magnetization, etc.) of ferrites, including ferrites doped by other elements, are determined by the synthesis method, surface condition, chemical composition, size, etc. For instance, coercivity is determined by ferrite-doping elements, such as Co, Ni, and Zn, and by the structures’ sizes [52,53,54,55,56]. In this case, coercivity can vary from units to hundreds of Oe. Superparamagnetic nanoparticles with almost zero coercivity and remanent magnetization are the most effective for drug delivery. These nanoparticles are the single-domain structures, sized 10–20 nm or less [57]. Nevertheless, authors [58] have shown the possibility of using microsized nanoparticles with significantly different magnetic parameters. It is worth mentioning that saturation magnetization of ferrites discussed in works above ranges from 7 to 100 umu/g depending on stoichiometry, size and shell.

Dipole interactions exerted in nanoparticles cluster have big impact on the magnetic nanoparticles’ applications. Dipole–dipole interactions determine the magnetic relaxation time, magnetic susceptibility, remanent magnetization and blocking temperature. These interactions depend on surface condition, and the functional shell which determines distance between magnetic cores [59,60]. Additionally, attention has been paid to the magnetic anisotropy presence in magnetic nanoparticles. For example, authors [61] have shown the effect of magnetic anisotropy presence on the efficiency of magnetic nanoparticles in magnetic hyperthermia applications.

**Figure 5:**
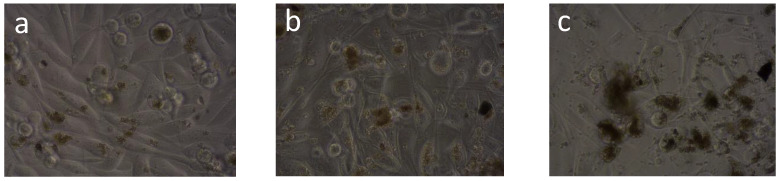
PC-3cells incubated with different concentrations of nanoparticles: 10 µg/mL (**a**), 50 µg/mL (**b**), 200 µg/mL (**c**).

The results of cytotoxic tests IC_50_ were collected in Table 4. The general morphology of the cell incubated for 72 h with different concentration of nanoparticles as well as nanoparticles behavior are shown in Figure 5 for PC-3cells. L929 and BxPC3 are presented in Figures S3 and S4.

The main cause of the cytotoxic effect of nanoparticles at the cellular level is due to their interaction with structures of vital cell such as membrane and mitochondria. The size, shape, type of shell and core strongly affect their pharmacokinetics such as distribution, metabolism, and absorption [62,63,64]. Grade of prion inflammatory and oxidative-stress–related cellular responses depend on the type of cell. The obtained results demonstrated that the toxic effect is the strongest for BxPC3 cells (IC_50_ 22.23 μg/mL). Cytotoxicity of Fe_3_O_4_-TMSPM-PAA/PALAm-Gd-PAA/PALAm–Carborane on HepG2 and human skin fibroblasts is low even at concentration of 1 mg/mL 72.5% and 90.2%, respectively, and IC50 cannot be calculated for these cell lines. Additionally, it should be noted that IC_50_ of 3-(isopropyl-o-carboranyl)hydrindone is 480 μg/mL (human skin fibroblasts), 332 μg/mL (HepG2) and 240 μg/mL MCF-7. The toxicity of carborane compound is incomparably small compared to the studied nanoparticles, although a greater sensitivity is also observed in cancer cell lines. It is also known that iron oxide nanoparticles modified by various methods have low toxicity [29,65]. In case Fe_3_O_4_/TEOS/TMSPM/GMA/Carborane the cytotoxicity was low and the IC_50_ was impossible to determine [29]. Thus, main toxic effect can come from Gd.

It is worth emphasizing that for Fe_3_O_4_/TEOS/TMSPM/GMA/Carborane as well as for Fe_3_O_4_-TMSPM-PAA/PALAm-Gd reduction in MCF-7 viability is significant. It shows the selective response of investigated types of nanoparticles on MCF-7. As suggested, *D. Alberti* MCF7 cells appear the better responding cells as expected on the basis of their higher Carbonic Anhydrase IX (CAIX) expression [66].

In Figure 5, PC-3cells incubated with different concentrations of nanoparticles are presented. It is demonstrates that, generally, nanoparticles are incorporated into the cell. At a higher concentration, the nanoparticles exhibit a tendency to aggregate. It is noticeable that only larger nanoparticles are unable to be incorporated into the cells; therefore, they aggregate more easily and are less toxic as a result.

Comparing the results obtained with those described in the literature, we can conclude the following: The amount of boron on iron oxide nanoparticles increased by 25.6 μg/g in comparison with the previously obtained results [28]. In our recent studies where only carboranes were immobilized, the content of boron was 20.7% and 15.3% [29]. The amount of boron on the carrier is an important parameter for improving the effectiveness of cancer treatment. Other authors suggest other methods of boron immobilization, such as coating with boron nitride nanospheres [67]. In this case, saturation magnetization was ~6 emu g^−1^, coercivity was ~22 Oe and remanent magnetization was 0.4 emu g^−1^. Gd is often being attached to nanoparticles, including Fe_3_O_4_ for magnetic field hyperthermia or magnetic resonance imaging [68,69,70]. Gd ions can be either introduced into the Fe_3_O_4_ structure [71] or immobilized with chelates, such as Gd-DTPA [30]. The simultaneous attachment of both Gd and B to magnetic nanocarriers for delivering NCT agents has been insufficiently studied; further research is required to identify a beneficial effect for cancer treatment by NCT.

## 3. Materials and Methods

### 3.1. Materials

Iron chloride (II) tetrahydrate, iron chloride (III) hexahydrate, AA, PALAm hydrochloride, aluminum oxide, TMSPM, ABAP, gadolinium (III) nitrate hexahydrate were purchased at Sigma Aldrich. Ethanol, methanol, benzene, *o*-xylol, hydrochloric acid, ammonium hydroxide aqueous solution, NaOH, and ethyl acetate were analytical grade.

### 3.2. Synthesis of Fe_3_O_4_ Nanoparticles (NPs)

Fe_3_O_4_ were synthesized by the precipitation method [72]. Briefly, to 100 mL of deionized water and 11.8 mL HCl (35%), 0.05 mol of FeCl_2_·4H_2_O and 0.1 mol of FeCl_3_·6H_2_O was added and intensively stirred. Then, 70 mL NH_4_OH was added drop by drop (pH > 9). The temperature was adjusted to 80 °C and reaction was going for 2 h. Then, procured nanoparticles were washed in deionized water several times before pH became neutral. After that NP were dried in Petry dish on air at 60 °C.

### 3.3. Functionalization of Fe_3_O_4_ NPs

Fe_3_O_4_ nanoparticles were modified according to Scheme 1.

#### 3.3.1. Double-Bond Formation on Fe_3_O_4_ NPs

The surface of nanoparticles was modified according to [73]. Briefly, in 100 mL of *o*-xylol, 1 g of Fe_3_O_4_ nanoparticles was added, then it was ultrasonicated for 2 h. After that 0.0126 mol of TMSPM was added to solution under mechanical stirring and heated to 80 °C. Reaction lasted for 5 h and then the product was washed 3 times with *o*-xylol and 2 times with diethyl ether.

#### 3.3.2. Thermoinitiated Graft Polymerization of Acrylic Acid

1g of functionalized Fe_3_O_4_ nanoparticles was dispersed in 90 mL of 50% aqueous ethanol solution by ultrasonication. Then, the solution was placed under argon flux. Then, 0.145 mol of acrylic acid was purified by filtering through the column with aluminum oxide and added to solution. After that, 0.25 mmol of ABAP was added. Reaction was kept at 80 °C for 24 h.

#### 3.3.3. Immobilization of PALAm

6 mmol of PALAm hydrochloride was neutralized by the reaction with 6 mmol of NaOH in 10 mL of MeOH. This reaction kept for 24 h at 60 °C. Then, PALAm solution was filtered from precipitate. Then, 40 mL of MeOH was added. In this solution 1 g of grafted nanoparticles was dispersed by ultrasonication for 2 h and then shaken in Test Tube Shaker Hei-MIX Multi Reax (Germany) for 24 h.

#### 3.3.4. Immobilization of Gd Ions

2 mmol of gadolinium (III) nitrate hexahydrate was dissolved in 50 mL of ethanol. Then, 1 g of nanoparticles was dispersed in this solution and shaken for 48 h.

#### 3.3.5. Immobilization of Polyelectrolytic Layer

Then, 0.3 g of PAA and 0.001 mol of Gd(NO_3_)_3_·6H_2_O was dissolved in 40 mL of ethanol. Then, 1g of nanoparticles was dispersed in this solution by ultrasonication for 30 min and then shaken for 1 h. After that nanoparticles were washed twice in ethanol and dispersed in a solution prepared as for immobilization of PALAm with added 1 mmol of Gd(NO_3_)_3_·6H_2_O by ultrasonication for 30 min, shaken for 1 h, washed twice in ethanol and dried.

### 3.4. Immobilization of Carborane Containing Agents

#### 3.4.1. Synthesis of 3-(Isopropyl-o-carboranyl) Hydrindone

**Scheme 2:**
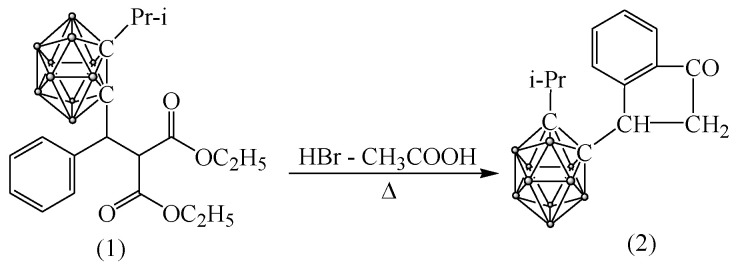
Production of 3-(isopropyl-o-carboranyl) hydrindone.

The interaction of carboranylmalonic ester (1) with a mixture of acetic and hydrobromic acids at 50–60 °C leads in good yield to 3-(isopropyl-o-carboranyl) hydrindone (2) (Scheme 2):

FTIR: 2800–3000 cm^−1^ (CH); 2600 cm^−1^ (BH); 1715 cm^−1^ (C=O); 1496, 1413, 1270 и 700 CМ^−1^ (vibrations of o-substituted benzene ring).

NMR H^1^ spectrum (DMSO), *J*, Hz: 7.79 (d., *J* = 7.6 Hz, 1H, C_sp2_H), 7.75 (d., *J* = 7,9 Hz, 1H, C_sp2_H), 7.68 (t., *J* = 7.4 Гц, 1H, C_sp2_H), 7.52 (t., *J* = 7.3 Hz, 1H, C_sp2_H), 4.16–3.98 (m., 1H, C-H), 3.15–2.92 (m., 2H, CH_2_), 2.63–2.44 (1H, m., CH(CH_3_)_2_), 1.39–1.35 (m., 3H, CH(CH_3_)_2_), 1.31–1.28 (m., 3H, CH(CH_3_)_2_), 1.65–2.4 (10H, m., BH).

#### 3.4.2. Immobilization of 3-(Isopropyl-o-carboranyl) Hydrindone

1 mmol of 3-(isopropyl-o-carboranyl) hydrindone was dissolved in 40 mL of ethyl acetate. Then, 1 g of nanoparticles was dispersed in solution for 1 h and shaken for 1 h. then it was twice washed in ethyl acetate and dried on air.

### 3.5. Methods of Characterization

FTIR spectrometer InfraLUM FT-08 was used to record FTIR spectra (range 400–4000 cm^−1^, 25 scans, 2 cm^−1^ resolution, in KBr pellets). Then, 7 mm KBr pellets were prepared by mixing of sample and KBr at weight ratio 0.006. Pellets were pressed with PIKE hand press (until No 12).

JEOL JSM-7500F scanning electron microscope was used for characterization of nanoparticles size and morphology.

EDX analysis was done using Hitachi TM 3030 with microanalysis system Bruker XFlash MIN SVE at 15 kV.

X-ray diffraction analysis was carried out on D8 ADVANCE ECO diffractometer (Bruker, Germany) using CuKα source (λ = 1.54060 Å). To identify the phases and study the crystal structure, the software BrukerAXSDIFFRAC.EVAv.4.2 and the international database ICDD PDF-2 were used.

The Mössbauer spectra were recorded on spectrometer MS1104Em operating in the regime of constant accelerations with a triangular shape of the Doppler velocity of the source relative to the absorber. As a source, ^57^Co nuclei in the Rh matrix were used. The spectra were measured at room temperature. Then, 0.1 g of the sample were placed in a uniform thin layer on a paraffin substrate and placed in a sample holder. The collection of spectra was carried out during the time required to achieve an effect of more than 1.5–2%. This quality of the collected spectra is due to covering of nanoparticles with silanes and polymers during the modification processes. The analysis of the obtained Mössbauer spectra was carried out using the SpectrRelax program code. A model consisting of two sextets characteristic of the states of iron oxide Fe_3_O_4_ in two sublattices A and B, which are characterized by different values of hyperfine magnetic fields, were used as a model for decoding the spectra.

DLS analyzes was provided with ZetaSizer Nano-ZS (Malvern, UK) for the estimating size. Then, 1 mg/mL water suspension with nanoparticles was used.

The investigation of macromagnetic properties was carried out using the vibrational magnetometer (the Liquid Helium Free High Field Measurement System (Cryogenic Ltd., London, UK). The measurements were implemented using the induction method, through a determination of the induced electromotive force of the induction in signal coils by a magnetized sample oscillating with a definite frequency at magnetic field B = ±1 T at 300 K temperature.

### 3.6. Colorimetric Essay

For the determination of concentration of carboxyl and amino groups colorimetric method was applied [73,74].

Concentration of carboxyl groups was examined using the toluidine blue. Nanoparticles were placed into 5 × 10^−4^ M toluidine blue solution, shaken for 3 h for providing maximum absorption. Then, samples were washed in NaOH solution (pH = 10) and twice in deionized water. Desorption was provided using 50% acetic acid for 10 min during vigorous shaking. Optical density of gained solution was determined at the wavelength 633 nm.

Concentration of amino groups was examined using acidic orange. Nanoparticles were placed into 5 × 10^−4^ M acidic orange solution and shaken for 12 h. then samples were washed in HCl solution (pH = 3). Desorption was provided using NaOH solution (pH = 12) for 15 min during vigorous shaking. Optical density of gained solution was determined at the wavelength 495 nm. Optical density was measured by Specord–250 spectrophotometer (Analytic Jena, Jena, Germany)

### 3.7. Cytotoxicity Assay

Cytotoxicity was evaluated using cancer cell lines: PC-3, BxPC3, MCF-7, HepG2 and normal cells: L929 and human skin fibroblasts. L929 PC3, epithelial cells of prostate cancer were cultured in DMEM-F12 medium with the addition of L-glutamine and 15 mM HEPES, with 10% FBS and 1% of antibiotics (penicillin-streptomycin) in temperature of 37 °C with 95% humidity and 5% CO_2_ flow. Pancreatic cancer cells, namely, BxPC-3 (primary pancreatic tumor) were cultured in RPMI-1640 medium supplemented with 10% fetal bovine serum (FBS), GlutaMAX™ and antibiotic-antimycotic. Breast cancer cells MCF-7 were cultured in Dulbecco’s Modified Eagle’s Medium (DMEM) supplemented with 10% fetal bovine serum (FBS), penicillin–streptomycin 1% (*v*/*v*). The cells were cultured in 5% CO_2_ incubator at 37 °C in a humidified atmosphere. Fibroblasts such as cells of L929 line obtained from mouse subcutaneous adipose tissue are recommended in the investigations of compounds cytotoxicity (PN-EN ISO 10993–5:2009 norm). L929 line cells were cultured in RPMI 1640 medium (with the addition of L-glutamine and 15 mM Hepes) with 10% FBS and 1% of antibiotics in temperature of 37 °C with 95% humidity and 5% CO_2_ flow.

The effect of Fe_3_O_4_ nanoparticles with immobilized gadolinium and boron isotopes on cell viability was determined using a quantitative colorimetric MTT assay [75,76]. The value of absorption is directly proportional to the number of living cells; hence, the reduction in tetrazolium salt into a colored product is possible only in cells with unaffected metabolism. For each concentration (in range from 1 to 1000 µg/mL) of the nanoparticles absorbance was measured spectrophotometrically at 570 nm on a microplate reader PowerWave XS (BioTek Instruments, Inc.) in three independent repetitions. Geometric mean of IC50 values (median inhibitory concentration—concentration required for 50% inhibition of cells viability compared to the negative control, which was accepted as 100%) were calculated by the GraphPad Prism (Version 7.0) statistical software.

## 4. Conclusions

This study introduces modified Fe_3_O_4_ nanocarriers as new potential agents for NCT based on the simultaneous delivery both of boron and gadolinium. Their hydrodynamic radius is 110 nm. The content of boron and gadolinium in the carrier is 0.077 mg/g and 0.632 mg/g, respectively. Though Mössbauer spectroscopy and XRD indicated changes in stoichiometry and structural defects due to oxidation, the magnetic properties were not altered considerably. The obtained nanoparticles exhibit visibly greater cytotoxicity in comparison with nanoparticles without immobilization of gadolinium. It is worth noting that toxicity is selective, and the most sensitive cell line is BxPC3. The most probable mechanism of the toxicity’s effect of nanoparticles seems to be oxidative stress. Proceeding from these data, it could be proposed that procured nanoparticles are applicable as NCT agents. The next goal of this study is research on distribution of nanocarriers in the body and its interaction with neutron flux.

## Figures and Tables

**Table 1:** Effect of concentration of initiator and monomer; time of the reaction on efficiency of PAA grafting.

	Concentration of Initiator, %			Concentration of Monomer, %			Time of Reaction, h		
	0.0125	0.03125	0.05	10	20	30	2	6	24
I_C=O_/I_Fe-O-Fe_	0.15	0.184	0.181	0.184	0.181	0.103	0.162	0.181	0.184

**Table 2:** Data on structural parameters of XRD analysis.

Sample	Lattice Parameter, Å	Crystalline Size, nm	Degree of Structural Ordering, %	Stoichiometry
Fe_3_O_4_	a = 8.29402	10.5	53.6	Fe_2.88_O_4_
Fe_3_O_4_-TMSPM	a = 8.31243	14.3	61.2	Fe_2.85_O_4_
Fe_3_O_4_-TMSPM-PAA	a = 8.29124	12.4	69.3	Fe_2.77_O_4_
Fe_3_O_4_-TMSPM-PAA/PALAm	a = 8.33838	18.7	63.1	Fe_2.76_O_4_
Fe_3_O_4_-TMSPM-PAA/PALAm-Gd	a = 8.33172	13.2	66.3	Fe_2.79_O_4_
Fe_3_O_4_-TMSPM-PAA/PALAm-Gd-PAA	a = 8.31174	11.4	66.1	Fe_2.79_O_4_
Fe_3_O_4_-TMSPM-PAA/PALAm-Gd-PAA/PALAm	a = 8.29846	12.6	66.2	Fe_2.69_O_4_
Fe_3_O_4_-TMSPM-PAA/PALAm-Gd-PAA/PALAm—Carborane	a = 8.30508	16.5	61.7	Fe_2.71_O_4_

**Table 3:** Experimental values of the Mössbauer spectra of Fe_3_O_4_ nanoparticles at different stages of modification and carboranes immobilization.

Sample	A-Sublattice H_n_, kOe	B-Sublattice H_n_, kOe	Intensity of Doublet, %	I_B-sublattice_/I_A-sublattice_	δ (Fe_3-δ_)O_4_	χ^2^
Fe_3_O_4_	475.34 ± 3.24	425.84 ± 4.53	0.7	74.9/24.4	0.327 ± 0.031	0.94 ± 0.03
Fe_3_O_4_-TMSPM	479.57 ± 2.32	427.07 ± 3.43	0.6	75.8/23.6	0.331 ± 0.025	1.09 ± 0.05
Fe_3_O_4_-TMSPM-PAA	469.42 ± 3.25	422.65 ± 4.31	1.2	84.5/14.3	0.330 ± 0.027	1.10 ± 0.06
Fe_3_O_4_-TMSPM-PAA/PALAm	465.75 ± 4.21	440.54 ± 3.64	2.5	87.4/10.1	0.332 ± 0.025	1.08 ± 0.04
Fe_3_O_4_-TMSPM-PAA/PALAm-Gd	465.32 ± 4.14	435.17 ± 5.42	1.6	86.5/11.9	0.322 ± 0.016	1.04 ± 0.10
Fe_3_O_4_-TMSPM-PAA/PALAm-Gd -PAA	459.65 ± 3.52	437.13 ± 2.31	1.1	87.2/11.7	0.304 ± 0.014	1.01 ± 0.09
Fe_3_O_4_-TMSPM-PAA/PALAm-Gd- PAA/PALAm	469.64 ± 2.45	435.65 ± 2.56	0.9	87.7/11.4	0.323 ± 0.021	1.05 ± 0.07
Fe_3_O_4_-TMSPM-PAA/PALAm-Gd- PAA/PALAm—Carborane	468.42 ± 2.63	437.39 ± 2.35	0.7	86.2/13.1	0.317 ± 0.027	1.21 ± 0.04

**Table 4:** Values of IC_50_ parameters for different cell lines.

Cell Line	IC_50_ Mean Values and 95%-Confidence Intervals (Parentheses) μg/mL
L 929	83.56 (72.36–96.42)
PC-3	80.63 (69.59–93.06)
BxPC3	22.23 (18.11–26.85)
MCF-7	55.32 (47.94–63.84)

## Data Availability

The data presented in this study are available on request from the corresponding author.

## Supplementary Materials

The following are available online at https://www.mdpi.com/article/10.3390/ijms22168687/s1.
