# Supplementary material for: Boron and Gadolinium Loaded Fe3O4 Nanocarriers for Potential Application in Neutron Capture Therapy
Source: Int J Mol Sci. 2021 Aug 13;22(16):8687. doi: 10.3390/ijms22168687 (PMC8395504; doi:10.3390/ijms22168687)
Supplement: Supplementary file 1 [file ijms-22-08687-s001.zip › ijms-1320906-supplementary.pdf]

Supplementary Material

For

Simultaneous Immobilization of B and Gd Isotopes on Fe3O4nanocarriers Modified with Polyelectrolytic Complexes for Potential Application in Neutron Cancer Therapy

Table S1. Data received from EDX analysis.

Sample	Elemental content, at. %								
	Fe	O	C	Si	N	Gd	Cl	B	Au
Fe_3O_4	35.7±0.3	60.8±0.3							3.4±0.6
Fe_3O_4-TMSPM	15±6.1	32.8±7.8	47.2±7.4	0.5±0.2					4.4±2.8
Fe_3O_4-TMSPM-PAA	20.7±4.1	42.8±1.7	30.3±1.9	0.7±0.2					3.4±0.5
Fe_3O_4-TMSPM-PAA/PALAm	28.5±3.6	36.9±3.3	27.2±0.1	0.1±0.2	2.9±0.9				3.7±0.3
Fe_3O_4-TMSPM-PAA/PALAm -Gd	15.5±3.8	41.1±3.6	24.3±1.15	0.6±0.2	8.5±2.5	2.9±0.85	0.03±0.05		6.8±1.7
Fe_3O_4-TMSPM-PAA/PALAm -Gd -PAA	21.43±1.62	27.7±2.4	36.8±1.2	0.7±0.2	7.4±0.75	0.3±0.1	-		0.7±0.2
Fe_3O_4-TMSPM-PAA/PALAm -Gd-PAA/PALAm	12±2	26.6±4.2	43.6±1.9	0.2±0	12±2	1.7±0.3	4.9±1.8		-
Fe_3O_4-TMSPM-PAA/PALAm -Gd-PAA/PALAm - Carborane	17.6±7.2	34.9±7	28.9±1.3	0.3±0.1	5.8±0.30	0.8±0.3	1.9±0.9	10.4±2.1	-

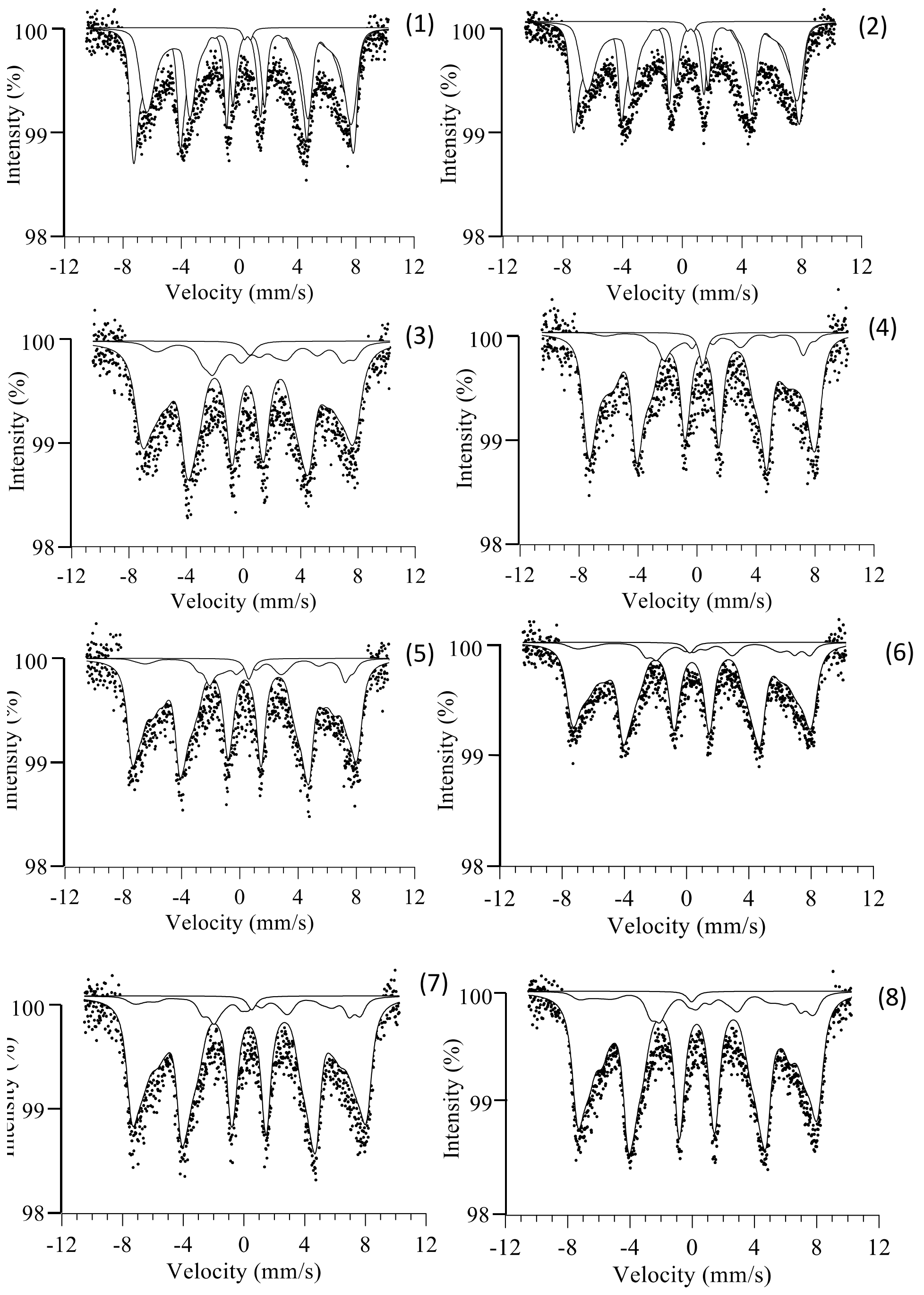

Figure S1. Mössbauer spectra of ^{57}Fe nuclei in Fe_3O_4 at different stages of modification: Fe_3O_4 (1), Fe_3O_4- TMSPM (2), Fe_3O_4-TMSPM-PAA (3), Fe_3O_4-TMSPM-PAA/PALAm (4), Fe_3O_4-TMSPM-PAA/PALAm-Gd (5), Fe_3O_4-TMSPM-PAA/PALAm-Gd -PAA (6), Fe_3O_4-TMSPM-PAA/PALAm-Gd- PAA/PALAm (7), Fe_3O_4-TMSPM-PAA/PALAm-Gd-PAA/PALAm – Carborane (8)

Table S2. Data of magnetic properties.

	**Magnetization B=1T, emu/g**	**Hc, Oe**	**Mr, emu/g**	**K**
Fe_3O_4	59.3	17.5	2.31	0.0392
Fe_3O_4- TMSPM	54.2	4.1	0.575	0.0114
Fe_3O_4-TMSPM-PAA	47.2	4.5	0.585	0.0116
Fe_3O_4-TMSPM-PAA/PALAm	48.4	4.05	0.502	0.0105
Fe_3O_4-TMSPM-PAA/PALAm-Gd	45.3	9.3	1.15	0.0247
Fe_3O_4-TMSPM-PAA/PALAm-Gd -PAA	46.2	9.97	1.1	0.0239
Fe_3O_4-TMSPM-PAA/PALAm-Gd-PAA/PALAm	43.8	8.5	0.845	0.0193
Fe_3O_4-TMSPM-PAA/PALAm-Gd-PAA/PALAm -Carborane	43.1	8.1	0.883	0.0205

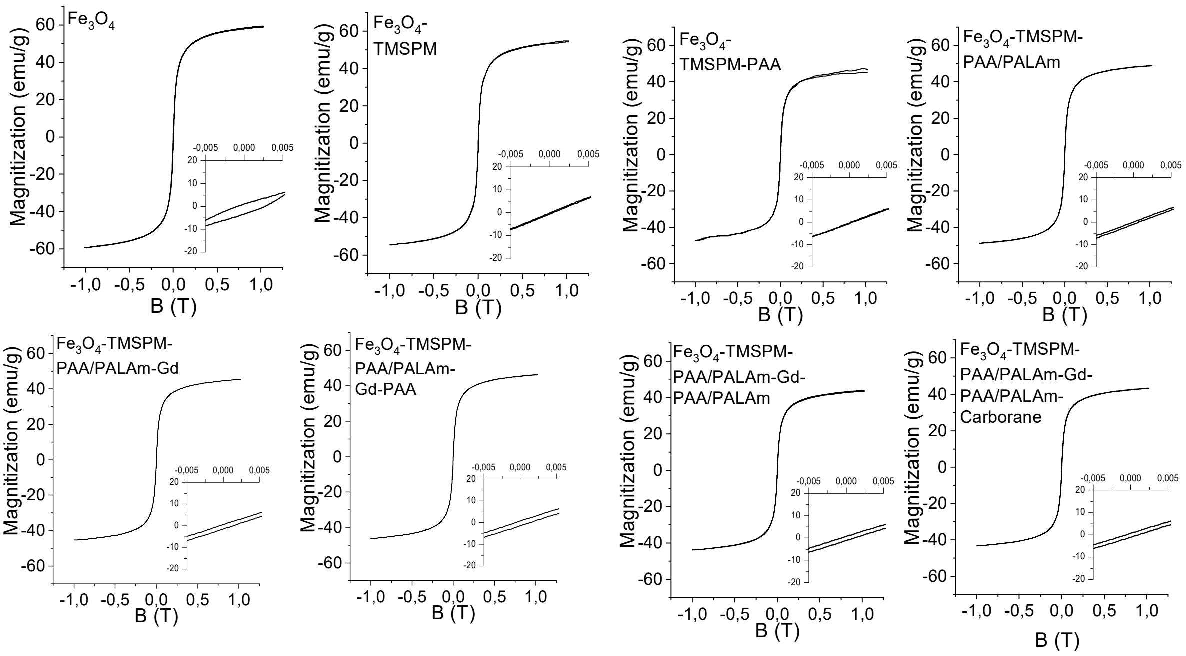

Figure S2. - Hysteresis loop of Fe_3O_4 at different stages of modification.

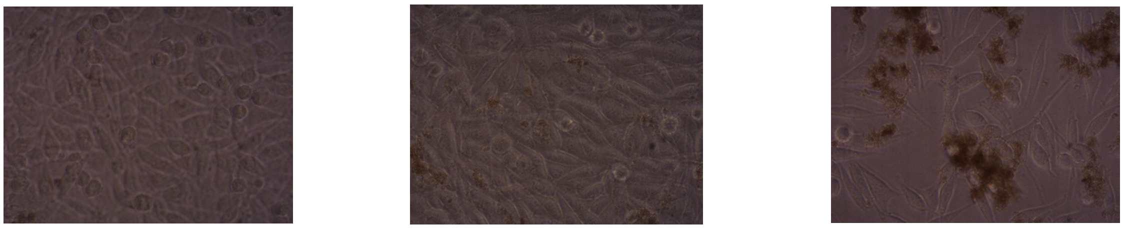

a b c

Figure S3. L929 cells incubated with different concentrations of nanoparticles a) 10µg/mL b) 50µg/mL c) 200µg/mL

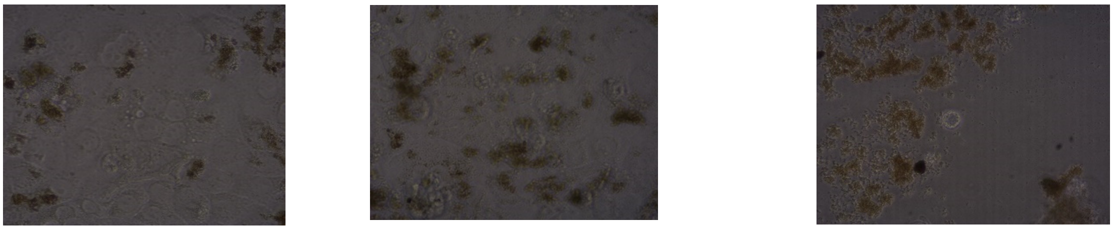

a b c

Figure S4. BxPC3 cells incubated with different concentrations of nanoparticles a) 80µg/mL b) 200µg/mL c) 500µg/mL
